# Return to play of young and adult professional athletes after COVID-19: A scoping review

**DOI:** 10.1016/j.jesf.2024.03.005

**Published:** 2024-03-18

**Authors:** Nicola Ceglie, Annamaria Petito, Giuseppe Cibelli

**Affiliations:** Department of Clinical and Experimental Medicine, University of Foggia, Foggia, Italy

**Keywords:** Return to play, Professional athletes, COVID-19, Scoping review

## Abstract

**Background/objective:**

Given the persistence of COVID-19 under various facets and mutations, there is an urgent need to understand the debate on a safe return to play for professional athletes (young and adults) recovering from the infection. This work offers a scoping and comprehensive review on the topic during the first two years of the pandemic event by providing an identification of main clusters of research, relevant gaps and significant insights for future investigation.

**Methods:**

The literature is selected using the search engines of: *PubMed®*, SCIENCEDIRECT, and SCOPUS. Further criteria for selection are: Time range of 2020–2022; Scope: Return to play of professional athletes recovering from COVID-19 infection; 3) Types of publications: Research papers, reviews, practice guidelines, case reports; 4) Language: English. Two independent researchers performed a quality check on a random sample (n = 30%) of publications.

**Results:**

Main results reveal four research clusters deepening the analysis on: myocarditis, cardiac diseases and return to play, training and rehabilitation, mass screening and risk assessment, and sport and bio-psycho-social sphere for a safe return to play. Major collaborations occur between UK-South Africa, UK-USA, USA-Canada, and USA-Australia.

**Conclusions:**

Important gaps refer to a lack of investigation on a safe return to play for female athletes in mostly all sports disciplines; on the other hand, sport and the bio-psycho-social sphere of the athlete is a fast-growing topic. Both deserve further attention in the immediate future to improve ad-hoc sport and exercise practices.

## 1 Introduction

In December 2019, a respiratory disease known as Coronavirus Disease-19 (COVID-19) broke out in Wuhan,^1^ and rapidly spread to become a pandemic declared by the World Health Organization,^2^ affecting economies and societies, including sporting events around the world, lasting through the subsequent years.^3^ Several lockdowns were adopted to limit the spread of the virus.^4^

As the pandemic progressed, the international debate grown rapidly to deepen the investigation of the effects of SARS-CoV-2 infection on athletes, including the provision of practice guidelines, rehabilitation and suggestions on a safe return to play (RTP).^5^, ^6^, ^7^, ^8^

This review focuses on understanding the main trends of the international debate on the effects of COVID-19 on RTP of young and adult professional athletes during the first two years of the pandemic (i.e. 2020-2022). Over this period of time, most review studies focused their attention on myocarditis and cardio-vascular diseases as a major result of SARS-CoV-2 infection, and the use of protocols and screenings before returning to play.^9^, ^10^, ^11^, ^12^, ^13^, ^14^, ^15^, ^16^, ^17^, ^18^ Nonetheless, other evident or latent diseases shall also be taken into account during the time intercourse between the illness and RTP. As a consequence, ad-hoc rehabilitation techniques and exercises should be considered as essential for an efficient RTP of the athlete. The rationale of the present work is to contribute to the research gap by uncovering hidden information on other diseases, other than myocarditis and cardio-vascular issues, and mapping their relationships occurring during the time intercourse for athletes from the illness to RTP.

Through a network analysis of keywords co-occurrence and a review of each cluster, the work aims at identifying main research lines, current gaps and scopes for new research topics deserving attention in sport and rehabilitation studies in the immediate future. This will help researchers, clinicians, athletes, and the whole sport entourages to deepen knowledge on RTP after COVID-19 with the adoption ad-hoc practices.

## 2 Methods

The study is performed in accordance with the guidelines of the Preferred Reporting Items for Systematic Reviews and Meta-Analyses (PRISMA).^19^^,^^20^

### 2.1 Equity, diversity, and inclusion in research

For the purpose of this study the recruitment of any participant is not applicable. The team of co-authors include a junior (male) researcher, and two senior academic professors (one male, one female). In addition, the quality check of the sample is performed by two (one female, one male) researchers.

### 2.2 Literature selection

The literature was selected through scientifically recognised search engines such as: PubMed®, SCIENCEDIRECT, and SCOPUS. Scopus presents a wider coverage of journals in comparison to Web of Science, while the latter includes a wider temporal coverage.^21^ Provided the short time range taken into account in this review, Web of Science is not considered for literature selection. PubMed® is the web portal of MEDLINE and includes additional features compared to the latter, such as for example ahead of print citations, or in-process citations. As for SCIENCEDIRECT, this database provides, other than social, life, physical sciences and engineering, health and medical references from the Elsevier company, the world largest scientific publisher.^22^, ^23^, ^24^ The search strategy is performed with advanced search builder interfaces which are present in PubMed®, SCIENCEDIRECT, and SCOPUS search engines and used Boolean operators ‘AND’ and ellipsis‘()’ in order to combine search terms referred to the query: ‘((COVID) AND (athletes) AND (return to play))’. In addition, the filter ‘Time range: 2020–2022’ has been applied.

### 2.3 Inclusion/exclusion

A first literature selection was performed with the use of R Project for Statistical Computing v. 4.3.0 (R software) and the library revtools (https://revtools.net/)^25^ to check for duplicates.

**Fig. 1:**
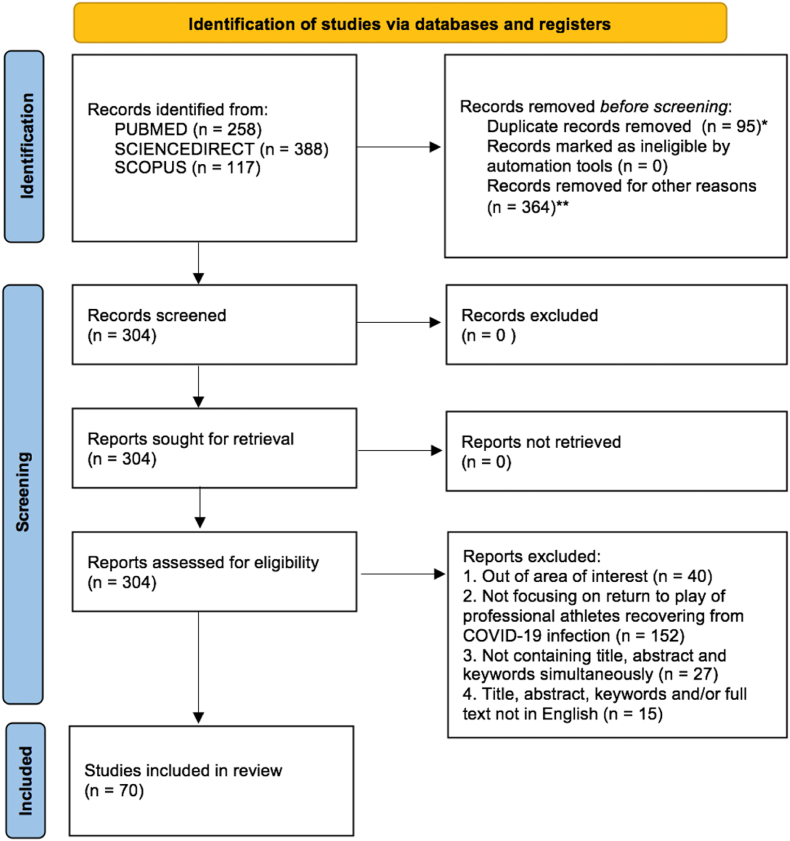
PRISMA Protocol for literature selection^19^^,^^20^ * Records automatically detected by R software and revtools library^25^ ** Records removed not falling within document types such as: Research articles, reviews (including meta-analysis and systematic reviews), case reports, practice guidelines.

Subsequently, one of the authors read titles, abstracts, keywords, and full-texts, and proceeded with selecting the records that: 1) did not focus on professional (young and/or adult) athletes and RTP from COVID-19. The definition of young athlete is provided by the Australian Institute of Sport^26^; 2) did not fall within the following types of documents: reviews, research articles, practice guidelines and case reports; 3) abstracts, titles, keywords were not present simultaneously. In terms of keywords, Medical Subject Heading (MeSH) keywords are considered as replacement of author's keywords when the latter are missing. MeSH is a subject heading list created by the National Library of Medicine (https://www.nlm.nih.gov/mesh/meshhome.html) and chosen by *PubMed®* indexers upon author's consensus on published articles; 4) abstracts, titles, keywords and/or full-texts were not in English. Two researchers from an academic institution performed a quality check (Supplement Table S1) for bibliometric analysis purposes on a random sample (n = 30%) of selected publications. In accordance with the PRISMA protocol (Fig. 1), the records that did not meet the selection criteria, were dropped. At the end of the selection phase the database comprised of 70 documents (Supplement Table S2).

### 2.4 Bibliometric analysis

VOSviewer v. 1.6.7^27^ was used to carry out the bibliometric analysis to obtain connection maps and co-occurrences among keywords (including MeSH keywords).^28^ In addition, R software and the library Bibliometrix (https://www.bibliometrix.org/home/) were also employed for descriptive statistics and risk of bias of missing items in data structure of bibliometric analysis^29^ (Supplement Table S3). The Bradford's Law^30^, ^31^, ^32^, ^33^ inspected the most productive journals and those obtaining the highest number of citations. The Lotka's Law^34^, ^35^, ^36^ and the Hirsch index explored the most productive authors.

The network analysis applied a bibliometric coupling approach^37^, ^38^, ^39^ with the full counting method.^40^^,^^41^ From a further inspection, non-relevant keywords (including those with no co-occurrences) were dropped,^42^^,^^43^ and a stemming and manual labelling procedure were employed.^42^, ^43^, ^44^ The visualization analysis^41^ employed with the algorithm by Waltman and van Eck^45^ provided the identification of keywords grouped within clusters.

## 3 Results

### 3.1 Source trends

During 2020–2022, the analysed documents were published in 47 journals with a publication range from 1 to 5 (Table 1). The Bradford's Law indicates 7 journals as core journals of Zone 1 (Table 1), whereas the remaining 17 and 23 journals appear in Zone 2 and 3, respectively. The top-10 most cited documents present a normalized local citation index range of 0.91–5.71 (Table 2).

**Table 1:** Bradford's Core and Zone 1 according to the number of documents.

Journals	Rank	Freq	Zone
*British Journal of Sports Medicine*	1	5	Zone 1
*International Journal of Environmental Research and Public Health*	2	5	
*Journal of Science and Medicine in Sport*	3	4	
*BMJ Open Sport & Exercise Medicine*	4	3	
*JACC-Cardiovascular Imaging*	5	3	
*JAMA Cardiology*	6	3	
*Clinical Journal of Sport Medicine*	7	2	
*Frontiers in Sports and Active Living*	8	2	Zone 2
*International Journal of Sports Medicine*	9	2	
*Orthopaedic Journal of Sports Medicine*	10	2	
*Scandinavian Journal of Medicine & Science In Sports*	11	2	
*Trends in Cardiovascular Medicine*	12	2	
*American Heart Journal Plus: Cardiology Research and Practice*	13	1	
*American Journal of Sports Medicine*	14	1	
*Annals of Medicine*	15	1	
*Applied Sciences-Basel*	16	1	
*Archives of Cardiovascular Diseases*	17	1	
*BMC Sports Science Medicine and Rehabilitation*	18	1	
*Canadian Journal of Cardiology*	19	1	
*Circulation*	20	1	
*Clinics in Sports Medicine*	21	1	
*Current Atherosclerosis Reports*	22	1	
*Current Emergency and Hospital Medicine Reports*	23	1	
*Current Sports Medicine Reports*	24	1	
*Current Treatment Options in Cardiovascular Medicine*	25	1	Zone 3
*European Cardiology Review*	26	1	
*European Heart Journal-Case Reports*	27	1	
*International Journal of Cardiology*	28	1	
*International Journal of Public Health*	29	1	
*Journal of Athletic Training*	30	1	
*Journal of Cardiovascular Development and Disease*	31	1	
*Journal of Clinical Medicine*	32	1	
*Journal of Sport Rehabilitation*	33	1	
*Journal of Sports Medicine and Physical Fitness*	34	1	
*Mayo Clinic Proceedings. Innovations, Quality & Outcomes*	35	1	
*Medicine & Science In Sports & Exercise*	36	1	
*Paediatric Respiratory Reviews*	37	1	
*Panminerva Medica*	38	1	
*Physician and Sports Medicine*	39	1	
*Physiological Reports*	40	1	
*Psychology of Sport and Exercise*	41	1	
*Psychoneuroendocrinology*	42	1	
*Rhode Island Medical Journal (2013)*	43	1	
*Science & Sports*	44	1	
*Sports Medicine*	45	1	
*Sports Medicine and Health Science*	46	1	
*Translational Sports Medicine*	47	1

**Table 2:** Top-10 most cited documents.

Document	Local Citations	Global Citations	LC/GC Ratio (%)	Normalized Local Citations	Normalized Global Citations
Kim J.H., 2021, *JAMA Cardiology*	24	122	19.67	5.71	3.98
Wilson M.G., 2020, *British Journal of Sports Medicine*	23	110	20.91	3.47	2.13
Brito D., 2021, JACC-*Cardiovascular Imaging*	19	113	16.81	4.52	3.69
Moulson N, 2021, *Circulation*	19	125	15.20	4.52	4.08
Martinez M.W, 2021, *JAMA Cardiology*	19	134	14.18	4.52	4.37
Phelan D., 2020, *JACC- Cardiovascular Imaging*	16	76	21.05	2.42	1.47
Stokes K.A., 2020, *International Journal of Sports Medicine*	6	52	11.54	0.91	1.01
Cavigli L., 2021, *International Journal of Cardiology*	6	29	20.69	1.43	0.95
Schwellnus M., 2021, British Journal of Sport Medicine	6	18	33.33	1.43	0.59

### 3.2 Influential authors and countries

**Fig. 2:**
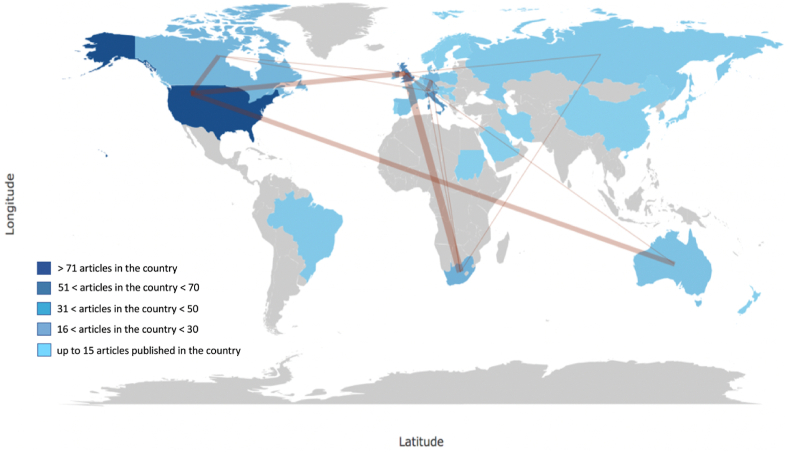
Countries' collaboration map. Notes: Min. edge = 2; Edge size = 5. The network of scientific collaboration across country is obtained by connecting a ‘manuscripts by authors’ matrix (https://www.bibliometrix.org/vignettes/Introduction_to_bibliometrix.html), whereas its magnitude is visualised through nodes (=authors) and their linkages (=co-authorships).^108^

A total of 573 co-authors were identified^34^, ^35^, ^36^; 88% (=506 co-authors) appeared with one paper, 10% (=55 co-authors) with 2 papers, and the remaining 2% (=12 authors) with 3–5 papers. The top-5 most prolific co-authors in terms of H-index were Baggish AL (=388 total citations) and Hull JH (=218 citations) both with an H-index = 4, followed by Kim JH, Martinez MW, and Phelan D (=332 citations; H-index = 3) (Supplement Fig. S1). The co-citation analysis revealed that co-authored publications derive from 22 countries worldwide. USA is the top-3 cited country with 857 total article citations (tac), followed by the UK (=225), and South Africa (=157) (Supplement Fig. S2). In addition, Fig. 2 depicts the cross-country collaboration map between authors. In particular, the blue and light blue colours represent the weight of collaboration according to the number of publications (including citations) of a particular country. The top-5 countries with the highest number of articles published are USA with 137 articles published, followed by UK, Germany, Italy, and France with 70, 41, 37, and 26 publications, respectively. The pink lines show the magnitude of collaboration between authors across countries; the thicker the line, the stronger is the magnitude. As a result, main collaborations occur between UK-South Africa, UK-USA, USA-Australia, and USA-Canada (Fig. 2).

### 3.3 Network analysis of keywords

**Fig. 3:**
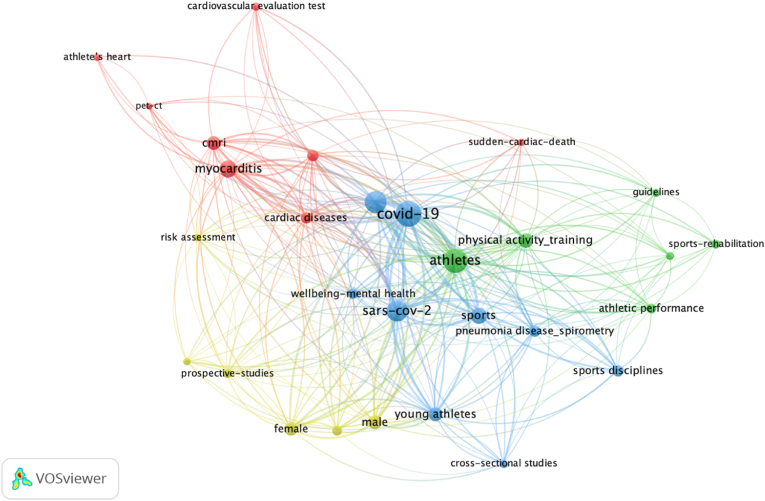
Bibliographic coupling of keywords. Layout: Attraction = 2.00; Repulsion = 1. Clustering: Resolution = 2; Min. Cluster size = 5.

Based on the number of co-occurrences among keywords, Fig. 3 shows main clusters of research as follows:

- Red cluster: Myocarditis, cardiac diseases and RTP
- Green cluster: Training and rehabilitation
- Yellow cluster: Mass screening and risk assessment
- Blue cluster: Sport and bio-psycho-social sphere.

### 3.4 Review of main clusters of research

A brief review for each identified cluster of research is carried out by grouping the selected literature into main sub-groups.

*Red cluster: Myocarditis, cardiac diseases and RTP.* Most studies refer to cardiac diseases, myocarditis, sudden cardiac death (SDC) and main diagnostic tests (particularly, cardiac magnetic resonance imaging - CMRI) for a safe RTP.^15^^,^^17^^,^^46^, ^47^, ^48^, ^49^, ^50^, ^51^, ^52^, ^53^, ^54^, ^55^ A further group of studies argues about the low incidence of COVID-19 effects related to cardiac injuries for competitive professional athletes.^15^^,^^50^^,^^56^^,^^57^ Current protocols also capture other long-term symptoms such as palpitations, chest pain, breathlessness, as well as fatigue and brain fog.^47^

Potential incidence of SCD also appears.^58^, ^59^, ^60^ Preliminary assessment with additional electrocardiogram (ECG) and echocardiogram (ECHO) before RTP is advised for athletes presenting mild to moderate COVID-19 symptoms.^59^^,^^61^ Daems et al.^52^ also suggest imaging techniques such as position emission tomography-computed tomography (PET-CT) with fluorodeossiglucosio (F-FDG), when CMRI demonstrates persistent myocardial oedema with late gadolinium enhancement (LGE). Other scholars^17^^,^^50^^,^^51^^,^^53^^,^^54^ point out to a thorough investigation of the myocarditis in terms of epidemiology, pathophysiology and clinical presentation of COVID-19. In terms of CMRI, multimodal imaging may be useful in the screening and clinical evaluation of athletes with suspected cardiovascular complications of the infection.^10^^,^^12^^,^^55^^,^^62^, ^63^, ^64^ CMR anomalies such as functional impairment, myocardial tissue abnormalities, late gadolinium elevation, or pericardial abnormalities fall in the range of 26–60% of the hospitalized athletes.^62^ This contrasts the evidence (range = 0–15.4%) based on CMRI performed from 10 to 194 days after the initial COVID-19 diagnosis.^12^^,^^63^ Maestrini et al.^64^ supports the efficacy of clinical assessment including exercise ECG. In addition, the diagnostic tests sub-group highlights the relevance of ECG and ECHO tests,^10^^,^^15^^,^^46^, ^47^, ^48^^,^^58^ whereas more specific diagnoses can be carried out through an in-depth cardiovascular assessment of the athlete^59^ including cardiopulmonary exercise tests (CPET)^65^, ^66^, ^67^ and 24 h Holter-ECG.^56^^,^^67^

*Green cluster: Training and rehabilitation*. This cluster includes works on physical activities and rehabilitation for a functional and safe recovery to RTP. Main sub-groups refer to physical activity-training, athletic performance, sport rehabilitation,^9^^,^^68^, ^69^, ^70^, ^71^, ^72^, ^73^, ^74^, ^75^, ^76^, ^77^, ^78^ and guidelines.^6^^,^^8^ Studies dealing with long-COVID effects are also included.^79^ Pillay et al.^68^ address the importance of adopting adequate RTP strategies to cover deficits in conditioning, strength, and neuromuscular proprioception. In their cross-sectional study, the authors also state that most of the interviewed athletes, during the illness period, trained at a daily basis in a range between 30 and 60 min, alone or with the help of a digital professional trainer. Most athletes also performed bodyweight strength training and cardio provided that they were equipped with steppers, bikes, or treadmills and functional exercises with the use of weights and elastics. In addition, proprioception (i.e. balance exercises), flexibility, and swimming pool training was used to favour muscle strength. Finally, the authors argue about the use of telemedicine to support rehabilitation at home. Stokes et al.,^69^ develop training strategies to mitigate potential losses due to prolonged disruption, including resistance training also with lighter loads (i.e. 10 weeks of knee-extension training at 30% and 80% of one repetition maximum, 12 weeks of whole body resistance, bench press and pull performances with one repetition maximum per 1 training session/week), plyometric training (i.e. concentric and eccentric muscle actions), exposure to running at high speed to ensure adequate muscle conditioning for a progressive RTP. Fabre et al.^9^ propose a careful approach to ensure the safety of athletes before RTP and optimize a rapid recovery of performance (for example, through countermovement and drop vertical jumps), through guidelines assessing fitness and health of athletes, deconditioning the physical abilities, and the monitoring of post-infection fatigue symptoms. A comprehensive cardiorespiratory-fitness (CRF) assessment is useful for screening residual damage of the myocardium and/or respiratory system.^70^ Specific recommendations are advised for a safe RTP and to prevent musculoskeletal injuries based on the individual situation of the athlete including pre-existing conditions, type of sport, intensity and length of the physical activity and risk of infection from other athletes (e.g. contact sports).^71^, ^72^, ^73^, ^74^ In addition, pulmonary workout for in- and expiratory muscles (with a daily exercise of at least 10 min for 6 weeks) to add resistance and flexibility during training, as well as strength training, such as 1 to 3 sets of exercises including 15–20 repetitions at an intensity that can well be accepted by the athlete, should be considered.^72^ As for severe cases such as heart failures, a respiratory rehabilitation of 2–3 sets of 10–15 repetitions in the range of 30%–60% of maximal inspiratory pressure should be included in the rehabilitation program. In addition, specific running workload (i.e. from low-to-high intensity running, high-speed running, and sprinting) of about 20%–40% than normal competitive periods can be advised for soccer players during an RTP spanning from 2 to 4 weeks.^73^ Finally, Martens et al.^74^ highlight the importance of telemedicine for home fitness and rehabilitation to ensure, among other workouts (e.g. weekly exercise program for muscle strengthening, core stability, balance, flexibility, neuromuscular co-ordination), vertical jump performance test and training purposes. The latter also include mental training with the use of virtual reality (VR).

Neuromuscular implications on movement and coordination are also present. Seligman et al.,^75^ focus on athletes' perception of their fitness capacity and trust in RTP after the infection. Main results suggest a reduction of 4 h/week in the total training activity. In this case, the athlete's perception is mainly associated to the total number of hours spent for sport activity, than trust for RTP or individual fitness capacity. Córdova-Martínez et al.^76^ address two key aspects: the impairment of performance and RTP. From clinical evidence, during the recovering phase, physical training and sports activity should be carried out progressively, both in terms of time and intensity. A 50% increase in intensity at the beginning of the recovering period and a gradual increase of 10–30% in the following weeks are suggested. Gattoni et al.^77^ show that general fatigue persisted for 77% of the sample, while muscle fatigue was perceived from 54% of players, respectively for 37 ± 28 and 38 ± 29 days after the illness. Therefore continuous monitoring such as deep analyses of medical history of the athlete, and low physical and psychological examinations, to avoid long term effects (e.g. persistent fatigue) is advised.^79^

*Yellow cluster: Mass screening and risk assessment.* This cluster highlights mass screening and risk assessment and methods of the analysed works (i.e. cohort-studies, and prospective-studies),^53^^,^^57^^,^^59^^,^^80^, ^81^, ^82^, ^83^, ^84^, ^85^ other than addressing the cases for male^52^^,^^70^^,^^77^^,^^85^ and female athletes.^78^, ^79^, ^80^, ^81^, ^82^, ^83^, ^84^, ^85^, ^86^, ^87^, ^88^

Daniels et al.^50^ underline the need for standardized times and interpretation of cardiac tests and the importance of CMRI in routine screening for a safe RTP. Krzywanski et al.^81^ analyse potential predictors of COVID-19 and antibody response using routine medical screening (i.e. physical exam, resting ECG, lab tests, troponin and antibody response) performed 12–68 days after the diagnosis of COVID-19. No further cardiac screening is required when athletes’ RTP is after two weeks without symptoms.^57^^,^^81^^,^^82^ Hull et al.^83^ investigate symptom duration and time loss in RTP. Main findings report that the most prevalent symptom is fatigue (57%), followed by dry cough (50%) and headache (46%). The median symptom duration (interquartile range, IQR) is 10 days (6–17), and 14% of the sample report symptoms >28 days, while the median time loss is 18 days (12–30), with 27% not fully available for training after 28 days from the initial date of infection.

The risk-assessment sub-group includes an in depth illustration of new recommendations from the Italian Federation of Sports Medicine, emphasising constant monitoring of health conditions and risk assessment.^7^ In addition, US scholars^5^^,^^10^^,^^15^^,^^51^ review American guidelines and recommendations and offer important guidance for risk assessment. The analyses generally split risk into categories according to the type of sport. The low-risk category includes sports such as bowling, horse riding, golf, swimming, tennis, and athletics. The medium-risk category considers baseball, softball, cross-country skiing, and gymnastics. Finally, the high-risk category includes basketball, football, ice hockey, lacrosse, and volleyball. The above categorization would provide better indications of risk-based strategies for athletes.^5^ Similarly, Niess et al.^84^ suggest that the identification and management of risk, other than the assessment of the infection on physical performance, provide the formulation of adequate recommendations about RTP at an evidence-based level.

The gender/sport sub-group addresses issues for male athletes: Stokes et al.^69^ consider professional rugby players in UK; Pedersen et al.^85^ focus on Danish elite soccer players; Milovancev et al.^70^ take into account Serbian volleyball athletes; Mehrsafar et al.^86^ consider Iranian professional footballers; Gattoni et al.^77^ provide an illustration on professional soccer players; and Daems et al.^52^ detail on elite football players. In contrast, few studies by De Sire et al.^78^ and Bruinvels et al.^88^ consider recommendations and requirements for female athletes.

*Blue cluster: Sport and bio-psycho-social sphere.* The Blue cluster brings together important relationships across the sub-groups: 1) well-being and mental health (i.e. anxiety, burnout, depression, loss of sleep, mindfulness, stress, fear of COVID-19, sedentary behaviour, competitive behaviour)^68^^,^^69^^,^^86^^,^^89^^,^^90^; 2) respiratory system (i.e. pneumonia)^70^^,^^77^^,^^87^ and cross-sectional studies^70^; 3) sports disciplines such as basketball, volley, football/rugby, and soccer.^46^^,^^52^^,^^68^^,^^69^^,^^77^^,^^78^^,^^85^^,^^86^^,^^91^, ^92^, ^93^

Pillay et al.^68^ highlight the contribution of the (negative) psychological consequences of COVID-19 such as the reported anxiety and stress in elite and semi-elite South African adult athletes. 50% of athletes were depressed, with loss of energy and lack of motivation to train and return to sport. Women were the hardest hit in all of these spheres, with potentially negative effects also on their mental health. Social isolation, reduction of physical exercise, sedentary behaviour and changes in diet led to negative psychological consequences affecting sleep quality and muscle fatigue. The authors stress the importance of telemedicine as main rehabilitation strategy. Mehrsafar et al.^86^ examine the relationship between anxiety competition, COVID-19 fear, and endocrine stress responses in professional soccer players after RTP on 90 Iranian male professional soccer players with an average age of 26.33 years. Significant positive correlations between COVID-19 anxiety and somatic competitive anxiety, cognitive competitive anxiety, and competitive response exist. In their study, the authors emphasise that copying strategies, mental consultation, and ad-hoc programs to reduce anxiety and fear are essential for mental rehabilitation. On the other hand, Myall et al.^89^ explore the relationships between awareness skills, resilience and athletic identity on anxiety and depression. Significant correlations were found between awareness, resilience, anxiety and depression. RTP was associated with a reduction in depression but not anxiety, while higher levels of awareness positively affected anxiety and depression. According to the authors, major rehabilitation strategies occur with a mindfulness approach. Levine et al.^90^ emphasise the understanding of factors influencing RTP and campus life of college athletes during the pandemic. The study reveals that confidence and motivation decreased stress and anxiety, and adaptive coping strategies influenced the athletes' experience of RTP during their campus life. Challenges associated with isolation and normal exercise routine are also evident.^9^ The study by Woods et al.,^94^ emphasises the existence of burnout symptoms for athletes affected by COVID-19 and forced to suspend their physical activity. The authors indicate that sport physiologists would play a critical role to strengthen social support. Brunweils et al.,^88^ on the other hand, is one of the few works focussing on symptoms and strategies for female athletes. The authors argue about the increase in phycological stress, anxiety, and lack of sleep, compared to their men's counterpart, and menstrual cycle alterations. The authors suggest menstrual cycle tracking, resting heart rate and sleeping monitoring, and telemedicine. In addition, anxiety may be a factor including a multitude of aspects able to extend and modify the training period. In turn, this could impact increasing injury risks when returning to play. A further psychological effect is the risk of compromising the ‘athletic identity’, due to a reduced ability to train, play and achieve goals, associated with feelings of loss, identity crisis and distress.

Finally, the sub-group related to sports disciplines include the majority of the literature, although the most representative disciplines appear basketball,^46^^,^^47^^,^^69^ volley,^70^^,^^78^ football/rugby,^68^^,^^69^^,^^80^^,^^89^ and soccer.^52^^,^^69^^,^^73^^,^^74^^,^^80^^,^^85^^,^^86^

## 4 Discussion

The debate on RTP after COVID-19 covers several fields. The four heterogeneous clusters of studies identified in this work provide a knowledge structure of RTP practices, guidelines and tools, other than understanding specific post-COVID effects which are essential to help sport teams and athletes with effective training, monitoring and rehabilitation exercises. In addition, during the first two years of the illness, the literature recognises major training time loss for recovery after COVID-19 infection. In particular, lost training occurs in the range of 7–51 days (median = 12 days)^73^^,^^95^; and 12–30 days (median = 18 days)^83^^1^ from different sport disciplines. Whereas, for athletes experiencing more severe effects such as myocarditis and cardiovascular symptoms or diseases, lost training occur in the range of 3–6 months^96^ (Table 3).

**Table 3:** COVID-19 and RTP relationships: Main features during 2020–2022.

COVID-19 and RTP relationships: Main features	Description	Authors
Symptoms	Common symptoms across all studies: Sore throat; chills; anosmia; ageusia; headache; cough; persistent-cough; fever; persistent-fever; conjunctivitis; shortness of breath; malaise; diarrhoea; nasal congestion; runny nose; dyspnoea; nausea; vomiting; loss of appetite; abdominal pain; myalgia; fatigue; severe fatigue; lethargy; chest pain; chest tightness; palpitations; arrhythmias; dizziness; light headedness; syncope; pneumonia; acute respiratory distress syndrome (ARDS), multiorgan failure.	Majority of studies in the sample
*Based on National Institute of Health (USA) classification*	- Class A1: asymptomatic/pauci-symptomatic athletes and athletes with ‘mild’ disease (anosmia, ageusia, headache, sore throat, fever, cough, malaise, mild upper respiratory tract illness, mild gastrointestinal illness); - Class A2: athletes with ‘moderate’ disease or athletes needing hospitalization (persistent fever, chills, myalgia, lethargy, dyspnoea, chest pain, chest tightness); - Class A3: athletes with ‘severe’ and/or ‘critical’ disease (respiratory failure, pneumonia, shock and multiorgan failure).	Casasco et al., 2022; de Abreu et al., 2022; Juhasz et al., 2022
*Based on extended versions of National Institute of Health (USA) classification*	- Post-acute COVID syndrome (PACS) for persistent symptoms after 3 weeks; - Chronic COVID after 12 weeks; - Long COVID or Post-Acute Sequelae of SARS-CoV-2 (PASC): The reported symptoms span a large breadth of cardiopulmonary and neurologic complaints including fatigue, palpitations, chest pain, cough, breathlessness, muscle soreness, myalgia, arthralgia, ventricular arrhythmias, decreased exercise tolerance, difficulty concentrating, anxiety, depression, brain fog, and dysautonomia including postural tachycardia syndrome (POTS); - POTS is characterized by change in heart rate with positional change, often accompanied by palpitations and decreased exercise tolerance.	Chilazi et al., 2021; Lindsay et al., 2021; Schwellnus et al., 2021; de Abreu et al., 2022; Niess et al., 2022; Snyders et al., 2022
*Based on neck check rule classification*	- Above neck symptoms: (runny/blocked nose or a tickly throat, coughing, sneezing, a sore throat); - Below neck symptoms: (high temperature, tight chest, persistent cough, nausea, vomiting, palpitations, malaise, diarrhoea, dyspnoea, muscle aches, joint pains, swollen lymph nodes).	Santos-Ferreira et al., 2020; Stokes et al., 2020; Mulcahey et al., 2021; Ross et al., 2021; Vaudreuil et al., 2021; Schmidt et al., 2022; Zhan et al., 2022
*Based on predominant anatomical focus classification*	- Upper respiratory (UR): sore throat, change in smell or taste or sinus problems reported, cough, fever. - Lower respiratory (LR): presence of dyspnoea, chest pain, cough, fever, other LR tract symptoms (e.g. wheeze). - Cough only: cough as the predominant symptom recorded and in the absence of coexisting dyspnoea and without other UR symptoms. - Gastrointestinal (GI): with predominant symptoms being diarrhoea, nausea, abdominal pain. - Non-specific: main clinical feature was fever, fatigue, headache, myalgia but a lack of any prominent respiratory or GI symptoms.	Hull et al., 2022
*Including cardiac involvement*	Shortness of breath; Dyspnoea; Fatigue; Palpitations; Dizziness; Chest pain; Light-headedness; Syncope; Myocardial injury; Heart failure; Cardiac arrhythmias (malignant arrhythmias); Thromboembolism; Pericarditis and myocarditis; SCD.	Augustine et al., 2021; Brito et al., 2021; Daniels et al., 2021; Fitzgerald et al., 2021; van Hattum et al., 2021; de Abreu et al., 2022; Giusto et al., 2022;
*Including neurological and psychological involvement*	Headache; Dizziness; Cognitive blunting; Brain fog; Dysautonomia; Altered consciousness; Altered sense of smell and taste; Neuromuscular involvement; Ischaemic stroke; Seizures; Encephalitis; Cranial neuropathies; Ocular muscle paralysis; Miller-Fisher syndrome; Guillain-Barré syndrome; Fear/anxiety; Insomnia; Stress/burnout; Depression.	Pillay et al., 2020; Stokes et al., 2020; Wilson et al., 2020; Bruinvels et al., 2021; Chilazi et al., 2021; Lindsay et al., 2021; Mehrsafar et al., 2021; Myall et al., 2021; Cordova et al., 2022; de Sire et al., 2022; Giusto et al., 2022; Levine et al., 2022; Woods et al., 2022
*Age-related symptoms and risks*	−18 and younger and 19–35: Asymptomatic and/or mild symptoms (see above); If chest pain, chest tightness, palpitations, shortness of breath exist, potential cardiovascular severe effects may appear such as myocarditis. The latter may also lead to SCD; - >35: Mild and/or moderate symptoms with co-morbidities (diabetes, hypertension, cardiovascular disease). The latter may also lead to higher risk of severe illness and/or death/SCD.	Augustine et al., 2021; Brito et al., 2021; Cavigli et al., 2021; Chilazi et al., 2021; Daniels et al., 2021; Erickson et al., 2021; Kim et al., 2021; Martinez et al., 2021; Moulson et al., 2021; Casasco et al., 2022
Cardiac assessment	Medical history; Physical examination; Blood tests (biomarkers, CRP, Troponin-I, cardiac Troponin-T, B-type natriuretic peptide, CBC, creatinine, ALT, AST, GGT, CPK, LDH, protein electrophoresis, D-dimer, ferritin, fibrinogen); ECG resting; ECG monitoring (24h/48 h ECG); Exercise ECG testing; Echocardiograph (Echo); Volume of oxygen consumption test (VO2); CPET; Maximal Exercise Test; CMR; Chest computed tomography (CT); EMB.	Phelan et al., 2020; Wilson et al., 2020; Augustine et al., 2021; Brito et al., 2021; Cavigli et al., 2021; Chilazi et al., 2021; Daniels et al., 2021; Erickson et al., 2021; Martinez et al., 2021; Moulson et al., 2021; Castelletti et al., 2022; Cordova et al., 2022; de Abreu et al., 2022; Hedon et al., 2022; Maestrini et al., 2022; Schmidt et al., 2022; Symanski et al., 2022; Tasca et al., 2022
Risk assessment		
*Minimizing the risk of transmission*	Appropriate hygiene and maximizing social distancing (6 feet); Use of masks (PPE); Rigorous monitoring and screening of symptoms; Widespread testing; Temperature checks; Comprehensive contact tracing (Bluetooth and GPS technology; Considerations for travel and facilities (asymptomatic travellers); Limiting or avoiding travel competition; Decreasing closeness of contact with other players (team, coaches, operational and medical staff) and duration of that contact; Cleaning equipment; Avoiding using shared food and drink, equipment, and spaces (e.g. locker rooms).	Santos-Ferreira et al., 2020; Stokes et al., 2020; Chiampas et al., 2021; Di Fiori et al., 2021; Fitzgerald et al., 2021; Milovancev et al., 2021; Mulcahey et al., 2021; Zhan et al., 2022
*Risk factors*	Older age; Cardiovascular-disease; Diabetes; Hypertension; Chronic Kidney Disease; Obesity; Ethnicity (Black, Asian, minority ethnic).	Augustine et al., 2021
*Risk related to sport*	- Low: Golf, Tennis [singles], Cricket, Skiing, Track and field with staggered starts, bowling, equestrian. - Medium: Gymnastics, Field hockey, Volleyball, Netball, softball, cross country, Baseball, Soccer/football, Swimming and diving competitions. - High: American football, Rugby Union, Rugby League, Australian Rules Football, Basketball, Wrestling, Lacrosse, Competitive cheerleading, Ice hockey.	Dove et al., 2020; Fitzgerald et al., 2021
*Injury risk and injury risk assessment*	- Risk of reduced performance as well as altered cardiovascular and muscle metabolic adaptations (reduce VO2max, reduce muscle strength and mass, reduce flexibility increase fat mass and increase risk of injury). As a result, muscle pain (myalgia) and fatigue are initial symptoms of the disease; - Open window: The period after intense bouts of exercise leading to increased inflammation, muscle damage, and an overall risk of infection. Re-conditioning period is important for recovery neuromuscular and cardiorespiratory functions to RTP without risk of injury. - Decline of efficiency of neuromuscular system, changes of body mass and composition and a consequent loss in terms of performance and endurance; - ACL injury in athletes: Neuromuscular activation patterns and the rate of recruitment of thigh muscle fibers, particularly quadriceps and hamstrings, play a key role in providing dynamic stability and reducing ACL injury risk; - Evaluation of neuromuscular control, coordination, flexibility, strength levels, and (im)balances not only allows the identification of an individual injury risk profile but also the building of individualized prevention programs such as: Muscle strengthening, flexibility, neuromuscular control, balance and agility, core stability, plyometrics, sport-specific movements, and endurance; - Specific equipment available treadmill, bicycles, balls, mats, skipping ropes, bands, and weights; - Mental training such as mental imagery and video analysis for technical and tactical sport-specific movements (e.g., using virtual reality tools); - Using remote-based tools for both evaluation and training purposes; - Continuous monitoring of both training load and athlete well-being to individually tailor optimal training (e.g., using specific wearable devices and portable technology); - Incorporating high-speed running into training. Beneficial to: hamstring architecture and sprint performance, and reducing injury risk; - Eccentric hamstring training and plyometric training.	Santos-Ferreira et al., 2020; Stokes et al., 2020; Martens et al., 2021; Mulcahey et al., 2021; Cordova et al., 2022; de Sire et al., 2022; Schimdt et al., 2022
RTP		
*What to do*	- Testing and monitoring in elite athletes (PCR-Testing; Antibody Testing; Social Contact; Monitoring of body temperature; Monitoring symptoms; Monitoring of Biomarkers; Cardiac Screening; Health screen of athletes prior to RTP); - Exercise training and injury risk (A graded re-implementation of sport-specific physical fitness training; Low-Moderate intensity aerobic training; Low-Moderate speed running; Treadmill, bicycle, rowing ergometer, resistance training equipment, body mass resistance circuit-based training, sports skills training (e.g. video analysis, virtual reality), personalized strength and conditioning training; muscle strengthening, flexibility, neuromuscular control, balance and agility, core stability, plyometrics; endurance; continuous monitoring of both training load); - Menstrual cycle monitoring; - Mental health (online platforms to monitoring mental health; psychological support; mindfulness; social support); - General health and well-being (recovery of sleep and nutrition; sleep monitoring; nutritional support; wellness monitoring to gauge fatigue and training adaptation); - Telehealth	Stokes et al., 2020; Bruinvels et al., 2021; Martens et al., 2021; Mulcahey et al., 2021; Ross et al., 2021; de Sire et al., 2022; Schmidt et al., 2022;
*Timeline*	- Day 1 (if tested positive for SARS-CoV-2): Athletes are required to self-isolate for 2 weeks; - Day 14 (if symptoms resolved or asymptomatic):Athletes are required to halt all sporting activities for 7 more days; - Day 21 (if asymptomatic or mildly symptomatic): Athletes can return to play gradually with evaluation of signs and symptoms; - Day 28 (if recovered after an illness that did not require hospitalization): Athletes are required to undergo physical exam and ECG before returning to Sports. Normal ECG - > Day 21; Abnormal ECG - > After Day 28; - After Day 28 (if recovered after sever illness that required hospitalization): Cardiac re-evaluation and rehab program are recommended; - Athletes with myocardial injury require a more comprehensive rehabilitation program with 3–6 months sport restriction.	Fabre et al., 2020; Cavigli et al., 2021; Daniels et al., 2021; Filomena et al., 2021; McKinney et al., 2021; van Hattum et al., 2021; Alosaimi et al., 2022; Daems et al., 2022; Klawitter et al., 2022; Mitrani et al., 2022; Patel et al., 2022; Schmidt et al., 2022; Symanski et al., 2022; Tasca et al., 2022
*Timeline based on European Association of Preventive Cardiology (EAPC)*	- Self-isolate for 7–14 days; - Refrain from exercise until symptom free for 7 days; - Clinical assessment in appropriate environment including blood tests (troponin and CRP); - If troponin positive, consider 12-lead ECG, echocardiogram, CMR and ECG monitor. If evidence of peri/myocarditis treatment is advised accordingly. - If no evidence of cardiac involvement, reassess after symptom free for 7 days and gradual return to training for an additional 7 days and return to normal training and/or play if asymptomatic and progressing well; - Repeat COVID-19 testing to ensure conversion to negative may be considered prior to return to training.	European Association of Preventive Cardiology (EAPC, 2020)
*Timeline based on Wilson* et al.*(2020)*	- Athletes with no signs or symptoms of COVID-19: Routine pre-participation medical evaluation; If no cardiac or respiratory investigations required, then RTP. - Athletes recovered from COVID-19: - If no symptoms, rest for 7 days and no sooner than day 10 from beginning of symptoms; - History + Physical Examination: Cardiac: 12-lead ECG and ECHO; - If normal results: gradual RTP ‘stop and reassess’ every 24 h; - If abnormal cardiac results: Cardiac MRI± 24 h ECG Holter; hs-cTnT and CPET; Training and regular respiratory restrictions, cardiac evaluation(s) and management according to current guidelines for myocarditis or other disorders. - Athlete NOT recovered from COVID-19 ≥ 14 from beginning of symptom (e.g. symptoms such as fatigue, cough, chest pain, palpitations, dyspnoea): - History + Physical Examination; Cardiac: 12-lead ECG and Cardiac MRI; Respiratory: CXR and lung function; Biochemistry: hs-cTnT, D-dimer and CRP; - If normal results: CPET and 24 h ECG Holter; - Normal results: gradual RTP ‘stop and reassess’ every 24 h; - Abnormal results: Training and regular respiratory restrictions, cardiac evaluation(s) and management according to current guidelines for myocarditis or other disorders. - Abnormal cardiac results: Training and regular respiratory restrictions, cardiac evaluation(s) and management according to current guidelines for myocarditis or other disorders. - Abnormal respiratory results: CT Chest ± CPET: - Normal results: gradual RTP ‘stop and reassess’ every 24 h; - Abnormal results: Training and regular respiratory restrictions, cardiac evaluation(s) and management according to current guidelines for myocarditis or other disorders; - Athlete hospitalized with COVID-19 (regardless of recovery, requires full cardiac and respiratory work up): - History + Physical Examination; Cardiac: 12-lead ECG and Cardiac MRI, 24 h ECG Holter and CPET; Respiratory: Repeat chest imaging and other measures as clinically indicated; Biochemistry: hs-cTnT, D-dimer & CRP: - Normal Results gradual RTP ‘stop and reassess’ every 24 h; - Abnormal Results:Training and regular respiratory restrictions, cardiac evaluation(s) and management according to current guidelines for myocarditis or other disorders.	

As for the reported main symptoms, there exist common symptoms recognised across all studies such as (Table 3): sore throat, chills, anosmia, ageusia, headache, cough, persistent-cough, fever, persistent-fever, conjunctivitis, shortness of breath, malaise, diarrhoea, nasal congestion, runny nose, dyspnoea, nausea, vomiting, loss of appetite, abdominal pain, myalgia, fatigue, severe fatigue, lethargy, chest pain, chest tightness, palpitations, arrhythmias, dizziness, light headedness, syncope, pneumonia, acute respiratory distress syndrome (ARDS), and multiorgan failure. Also, several symptom related classifications are found in the literature terms of (Table 3): asymptomatic/pauci-symptomatic athletes and athletes with ‘mild’ diseases (anosmia, ageusia, headache, sore throat, fever, cough, malaise, mild upper respiratory tract illness, mild gastrointestinal illness); athletes with ‘moderate’ disease or athletes needing hospitalization (persistent fever, chills, myalgia, lethargy, dyspnoea, chest pain, chest tightness); athletes with ‘severe’ and/or ‘critical’ disease (respiratory failure, pneumonia, shock and multiorgan failure).^54^^,^^87^^,^^97^ According to works considering extended versions of the symptom classification provided by the National Institute of Health (USA), main symptom clusters are as follows (Table 3): Post-acute COVID syndrome (PACS) for persistent symptoms after 3 weeks; Chronic COVID after 12 weeks; Long COVID or Post-Acute Sequelae of SARS-CoV-2 (PASC); Postural tachycardia syndrome (POTS).^47^^,^^54^^,^^79^^,^^84^^,^^98^^,^^99^ Other studies, instead, consider ‘above neck symptoms’ such as runny/blocked nose or a tickly throat, coughing, sneezing, a sore throat; and ‘below neck symptoms’ such as: high temperature, tight chest, persistent cough, nausea, vomiting, palpitations, malaise, diarrhoea, dyspnoea, muscle aches, joint pains, swollen lymph nodes^8^^,^^17^^,^^69^^,^^96^^,^^100^, ^101^, ^102^ (Table 3). The study by Hull et al.^83^ takes into account a symptom-related classification based on: Upper respiratory (UR); Lower respiratory (LR); Cough only; Gastrointestinal (GI); and Non-specific symptoms including, for example, fatigue, headache, or myalgia. Finally, other studies consider a symptom classification based on cardiac^46^^,^^48^^,^^50^^,^^54^^,^^103^^,^^104^ and neurological and psychological involvement.^47^^,^^58^^,^^68^^,^^69^^,^^76^^,^^79^^,^^86^^,^^88^, ^89^, ^90^^,^^94^^,^^104^^,^^105^

In terms of age-related symptoms and risks, the majority of studies recognise the following (Table 3):

− 18 and younger and 19–35: Asymptomatic and/or mild symptoms. Whereas chest pain, chest tightness, palpitations, shortness of breath exist, potential cardiovascular severe effects may appear such as myocarditis. The latter may also lead to SCD.^15^^,^^46^^,^^47^^,^^49^^,^^50^^,^^59^^,^^66^^,^^80^^,^^97^
- >35: Mild and/or moderate symptoms with co-morbidities (diabetes, hypertension, cardiovascular disease). The latter may also lead to higher risks of severe illness and/or death/SCD.^47^^,^^48^^,^^59^^,^^97^

According to the relationship occurring between RTP and risk assessment, the majority of works in the studied sample consider proper ways to minimise the risk of contracting the illness through the use of (Table 3): Appropriate hygiene and social distancing (6 feet), masks (PPE), rigorous monitoring and screening of symptoms, widespread testing, temperature checks, contact tracing (through digital devices), cleaning equipment, ad-hoc facilities (for asymptomatic travellers), or limiting or avoiding travel competition, decreasing the duration of contacts with other players, teams, coaches, operational and medical staff, and avoiding using shared food and drink and equipment.^8^^,^^69^^,^^70^^,^^96^^,^^102^^,^^103^^,^^106^^,^^107^ Other studies^8^^,^^17^^,^^69^^,^^74^^,^^76^^,^^96^^,^^105^ consider injury and injury risk assessment in terms of (Table 3): Risk of reduced performance as well as altered cardiovascular and muscle metabolic adaptations; Open window, including re-conditioning timings for the recovery of neuromuscular and cardiorespiratory functions; Decline of efficiency of neuromuscular system, changes of body mass and composition and a consequent loss in terms of performance and endurance; ACL injury in athletes for which quadriceps and hamstrings play a key role in providing stability; Evaluation of neuromuscular control, coordination, flexibility, strength levels, and (im)balances; Specific equipment such as: treadmill, bicycles, balls, mats, skipping ropes, bands, and weights; Mental training; Use of remote-based tools for both evaluation and training purposes; Continuous monitoring of both training load and athlete well-being to individually tailor optimal training; High-speed running; and eccentric hamstring and plyometric training.

As for RTP, the majority of studies^8^^,^^17^^,^^69^^,^^74^^,^^88^^,^^100^^,^^105^ recognise: Testing and monitoring in elite athletes; Exercise training and injury risk; Menstrual cycle monitoring; Mental health; General health and well-being; and Telemedicine (Table 3).

The investigation highlights the need towards further studies considering physical or mental rehabilitation after COVID particularly relevant for female athletes. For this category of athletes the literature still fails to capture important insights. On the other hand, relevant understandings derive from studies capturing the bio-psycho-social sphere for a safe RTP. This arena of investigation involves both psychological and cognitive rehabilitation practices for the single athlete but also his/her own behaviours (explicit or latent) and the relationship occurring across the whole entourage of medical staff, coaches, family and friends. The contribution of changes in the psychological sphere is as challenging and important aspect as cardiac and pulmonary effects. Psychologists and counsellors play a crucial role in helping athletes to cope with these challenges, providing support, and teaching coping strategies to manage stress and trauma. Similarly, cognitive rehabilitation helps athletes to regain cognitive functions and adapt to any long-term change from the infection. Further research also into this direction will contribute to close the current literature gap and adequately address a thorough recovery management of the athlete.

### 4.1 Limitations and future insights

This work is not without limitations. First, despite using top scientific databases, some publication biases may have occurred. These span from potential biased citation analysis to biased indicators of research impact. Second, articles or other related documents published in journals with different features from those requested or indexed in the considered databases may have been excluded. Future research should consider extending the current analysis by including papers and other documents from other databases, including works also published in other languages than English.

## 5 Conclusion

This review captures the main international debate on a safe RTP for athletes in the first two years of the pandemic event. The investigation provided evidence of a fast growing research touching relevant aspects such as cardiac and respiratory effects, other than proprioception and neuromuscular impacts, the need to follow proper training and exercises, and the importance to harmonise guidelines and practices across relevant sports disciplines and countries. Two relevant gaps should be filled in the incoming years. First, a better understating of COVID-19 impacts (and the effects of rehabilitation practices) on female athletes.

Based on this insights, future studies should also point towards testing the existence of differences on the effects of COVID-19 on RTP between and within groups of males and females and adults and young athletes across sports disciplines; as well as testing the differences between and across the above groups of ad-hoc rehabilitation practices for the recovery of the illness. Finally, an investigation based on a detailed recovery management of the athlete including ad-hoc sport and training techniques other than phyco-cognitive therapies is advised for a future debate.

## Author contributions

NC provided: Conceptualization; Data curation; Investigation; Methodology; Software; Validation; Visualization; Writing - original draft; Writing - Review & editing; AP and GB provided: Supervision; Resources. All authors approved the submitted version.

## Ethics committee

No formal approval was necessary for this study.

## Funding

We acknowledge funding from the PhD in Neuroscience and Education, 37 cycle, 10.13039/100016112University of Foggia (Italy).

## Declaration of competing interest

The authors declare that they have no known competing financial interests or personal relationships that could have appeared to influence the work reported in this paper.

## Footnotes

1 Data refers to professional athletes from different UK sport institutes.^83^
