# Supplementary material for: Return to play of young and adult professional athletes after COVID-19: A scoping review
Source: J Exerc Sci Fit. 2024 Mar 18;22(3):208–20. doi: 10.1016/j.jesf.2024.03.005 (PMC10973587; doi:10.1016/j.jesf.2024.03.005)
Supplement: Multimedia component 1 [file mmc1.docx]

**Supplementary material**

| 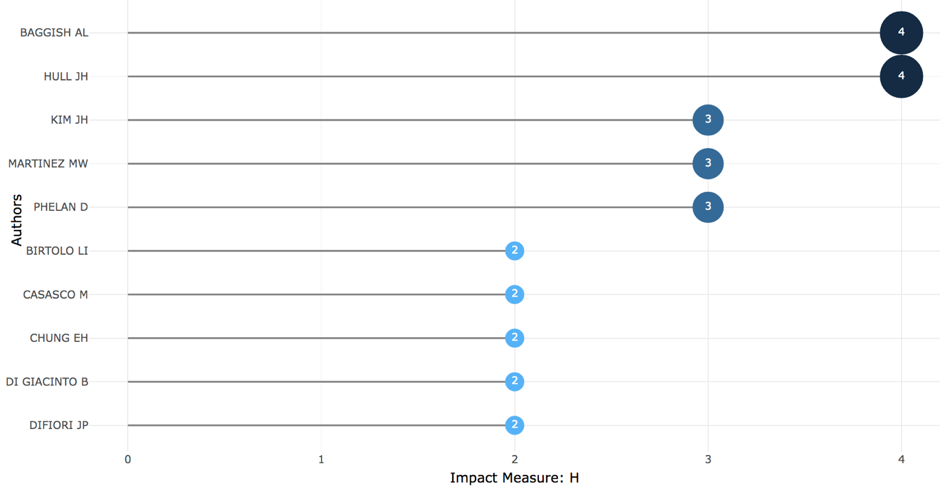 |
| --- |
| Figure S1. Influential authors |

| 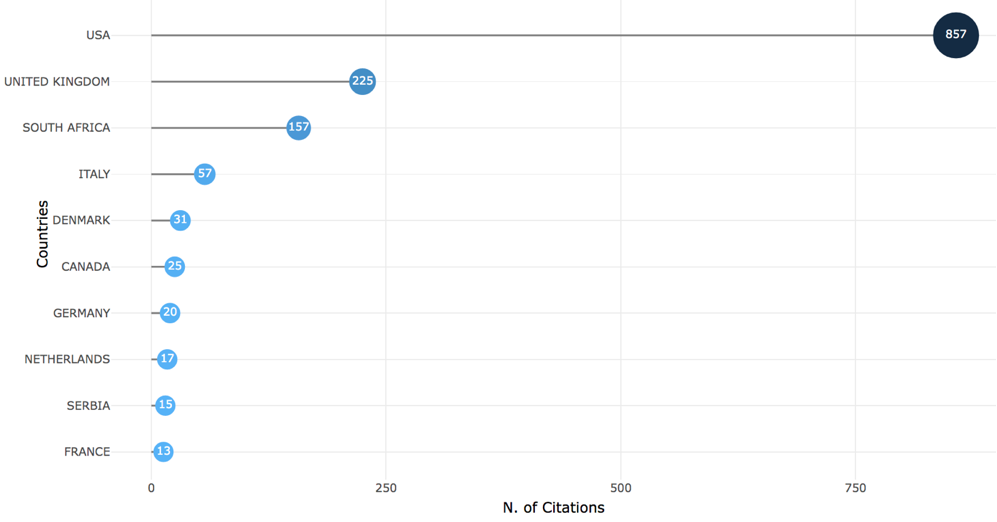 |
| --- |
| Figure S2. Top-10 cited countries |

Table S1: Quality check for bibliometric analysis purposes

| Source engine (please tick): | *PubMed®* | SCIENCEDIRECT | | SCOPUS |
| --- | --- | --- | --- | --- |
|  |  |  | |  |
| *Eligibility criteria* |  |  | |  |
| 1. Are the terms COVID-19, return to play (RTP) and athlete(s) present in the title, abstract or keywords? | Yes | | No | |
| 2. Is the study published in the time range 2020-2022? | Yes | | No | |
| 3. Are the titles, keywords, and abstracts present simultaneously in the publication? | Yes | | No | |
| 4. Are the titles, keywords, abstracts and full-texts written in English? | Yes | | No | |
| 5. Does the study fall within the document types articles, reviews, case reports and guidelines? | Yes | | No | |

Table S2. Selected publications in the studied work

| Authors | Title | Journal/Book | Year |
| --- | --- | --- | --- |
| Alosaimi B, et al. | Cardiovascular complications and outcomes among athletes with COVID-19 disease: a systematic review | BMC Sports Sci Med Rehabil | 2022 |
| Augustine DX, et al. | Coronavirus Disease 2019: Cardiac Complications and Considerations for Returning to Sports Participation | Eur Cardiol | 2021 |
| Brito D, et al. | High Prevalence of Pericardial Involvement in College Student Athletes Recovering From COVID-19 | JACC Cardiovasc Imaging | 2021 |
| Bruinvels G, et al. | COVID-19-Considerations for the Female Athlete | Front Sports Act Living | 2021 |
| Casasco M, et al. | Return to Play after SARS-CoV-2 Infection in Competitive Athletes of Distinct Sport Disciplines in Italy: A FMSI (Italian Federation of Sports Medicine) Study | J Cardiovasc Dev Dis | 2022 |
| Castelletti S, et al. | The Athlete after COVID-19 infection: what the scientific evidence? What to do? | Panminerva Med | 2022 |
| Cavigli L, et al. | A prospective study on the consequences of SARS-CoV-2 infection on the heart of young adult competitive athletes: Implications for a safe return-to-play | Int J Cardiol | 2021 |
| Chiampas GT, Ibiebele AL | A Sports Practitioner's Perspective on the Return to Play During the Early Months of the COVID-19 Pandemic: Lessons Learned and Next Steps | Sports Med | 2021 |
| Chilazi M, et al. | COVID and Cardiovascular Disease: What We Know in 2021 | Curr Atheroscler Rep | 2021 |
| Córdova-Martínez A, et al. | Effects and Causes of Detraining in Athletes Due to COVID-19: A Review | Int J Environ Res Public Health | 2022 |
| Daems JJN, et al. | Case report: the role of multimodal imaging to optimize the timing of return to sports in an elite athlete with persistent COVID-19 myocardial inflammation | Eur Heart J Case Rep | 2022 |
| Daniels CJ, et al. | Prevalence of Clinical and Subclinical Myocarditis in Competitive Athletes With Recent SARS-CoV-2 Infection: Results From the Big Ten COVID-19 Cardiac Registry | JAMA Cardiol | 2021 |
| Davey MS, et al. | Return to Play Following COVID-19 Infection-A Systematic Review of Current Evidence | J Sport Rehabil | 2022 |
| de Abreu RM. | Cardiac Changes Related to COVID-19 in Athletes: A Brief Review | Curr Emerg Hosp Med Rep | 2022 |
| De Sire A, et al. | Neuromuscular Impairment of Knee Stabilizer Muscles in a COVID-19 Cluster of Female Volleyball Players: Which Role for Rehabilitation in the Post-COVID-19 Return-to-Play? | Appl Sci (Basel) | 2022 |
| Diamond AB, et al. | Interim Guidance on the Preparticipation Physical Examination for Athletes During the SARS-CoV-2 Pandemic | Clin J Sport Med | 2021 |
| DiFiori JP, et al. | Return to sport for North American professional sport leagues in the context of COVID-19 | Br J Sports Med | 2021 |
| Dove J, et al. | COVID-19 and Review of Current Recommendations for Return to Athletic Play | R I Med J (2013) | 2020 |
| Erickson JL, et al. | Use of Electrocardiographic Screening to Clear Athletes for Return to Sports Following COVID-19 Infection | Mayo Clin Proc Innov Qual Outcomes | 2021 |
| Fabre JB, et al. | Managing the combined consequences of COVID-19 infection and lock-down policies on athletes: narrative review and guidelines proposal for a safe return to sport | BMJ Open Sport Exerc Med | 2020 |
| Filomena D, et al. | The role of cardiovascular magnetic resonance in the screening before the return-to-play of elite athletes after COVID-19: utility o futility? | J Sports Med Phys Fitness | 2021 |
| Fitzgerald HT, et al. | Covid-19 and the impact on young athletes | Paediatr Respir Rev | 2021 |
| Gattoni C, et al. | COVID-19 disease in professional football players: symptoms and impact on pulmonary function and metabolic power during matches | Physiol Rep | 2022 |
| Giusto E, Asplund CA | Persistent COVID and a Return to Sport | Curr Sports Med Rep | 2022 |
| Goergen J, et al. | COVID-19: the Risk to Athletes | Curr Treat Options Cardiovasc Med | 2021 |
| Halle M, et al. | Exercise and sports after COVID-19-Guidance from a clinical perspective | Transl Sports Med | 2021 |
| Hédon C, et al. | Cardiac screening before returning to elite sport after SARS-CoV-2 infection | Arch Cardiovasc Dis | 2022 |
| Hull JH, et al. | Clinical patterns, recovery time and prolonged impact of COVID-19 illness in international athletes: the UK experience | Br J Sports Med | 2022 |
| Juhász V, et al. | Short and mid-term characteristics of COVID-19 disease course in athletes: A high-volume, single-center study | Scand J Med Sci Sports | 2022 |
| Kim JH, et al. | Coronavirus Disease 2019 and the Athletic Heart: Emerging Perspectives on Pathology, Risks, and Return to Play | JAMA Cardiol | 2021 |
| Klawitter P, et al. | Low Risk of Cardiac Complications in Collegiate Athletes After Asymptomatic or Mild COVID-19 Infection | Clin J Sport Med | 2022 |
| Krzywański J, et al. | Elite athletes with COVID-19 - Predictors of the course of disease | J Sci Med Sport | 2022 |
| Krzywański J, et al. | Vaccine versus infection - COVID-19-related loss of training time in elite athletes | J Sci Med Sport | 2022 |
| Lemes IR, et al. | Acute and post-acute COVID-19 presentations in athletes: a systematic review and meta-analysis | Br J Sports Med | 2022 |
| Levine O, et al. | The Collegiate Athlete Perspective on Return to Sport Amidst the COVID-19 Pandemic: A Qualitative Assessment of Confidence, Stress, and Coping Strategies | Int J Environ Res Public Health | 2022 |
| Lindsay RK, et al. | What are the recommendations for returning athletes who have experienced long term COVID-19 symptoms? | Ann Med | 2021 |
| Löllgen H, et al. | Recommendations for return to sport during the SARS-CoV-2 pandemic | BMJ Open Sport Exerc Med | 2020 |
| Maestrini V, et al. | Systematic Cardiovascular Screening in Olympic Athletes before and after SARS-CoV-2 Infection | J Clin Med | 2022 |
| Maestrini V, et al. | Low prevalence of cardiac abnormalities in competitive athletes at return-to-play after COVID-19 | J Sci Med Sport | 2022 |
| Martens G, et al. | Exercise-Based Injury Prevention in High-Level and Professional Athletes: Narrative Review and Proposed Standard Operating Procedure for Future Lockdown-Like Contexts After COVID-19 | Front Sports Act Living | 2021 |
| Martinez MW, et al. | Prevalence of Inflammatory Heart Disease Among Professional Athletes With Prior COVID-19 Infection Who Received Systematic Return-to-Play Cardiac Screening | JAMA Cardiol | 2021 |
| McKinney J, et al. | COVID-19-Myocarditis and Return to Play: Reflections and Recommendations From a Canadian Working Group | Can J Cardiol | 2021 |
| Mehrsafar AH, et al. | Competitive anxiety or Coronavirus anxiety? The psychophysiological responses of professional football players after returning to competition during the COVID-19 pandemic | Psychoneuroendocrinology | 2021 |
| Milovancev A, et al. | Cardiorespiratory Fitness in Volleyball Athletes Following a COVID-19 Infection: A Cross-Sectional Study | Int J Environ Res Public Health | 2021 |
| Mitrani RD, et al. | Utility of exercise testing to assess athletes for post COVID-19 myocarditis | Am Heart J Plus | 2022 |
| Mitrani RD, et al. | Long-term cardiac surveillance and outcomes of COVID-19 patients | Trends Cardiovasc Med | 2022 |
| Moulson N, et al. | SARS-CoV-2 Cardiac Involvement in Young Competitive Athletes | Circulation | 2021 |
| Mulcahey MK, et al. | Sports Medicine Considerations During the COVID-19 Pandemic | Am J Sports Med | 2021 |
| Myall K, et al. | Anxiety and Depression during COVID-19 in Elite Rugby Players: The Role of Mindfulness Skills | Int J Environ Res Public Health | 2021 |
| Niess AM, et al. | COVID-19 in German Competitive Sports: Protocol for a Prospective Multicenter Cohort Study (CoSmo-S) | Int J Public Health | 2022 |
| Patel P, Thompson PD | Diagnosing COVID-19 myocarditis in athletes using cMRI | Trends Cardiovasc Med | 2022 |
| Pedersen L, et al. | Reopening elite sport during the COVID-19 pandemic: Experiences from a controlled return to elite football in Denmark | Scand J Med Sci Sports | 2021 |
| Petersen SE, et al. | Cardiovascular Magnetic Resonance for Patients With COVID-19 | JACC Cardiovasc Imaging | 2022 |
| Phelan D, et al. | Screening of Potential Cardiac Involvement in Competitive Athletes Recovering From COVID-19: An Expert Consensus Statement | JACC Cardiovasc Imaging | 2020 |
| Pillay L, et al. | Nowhere to hide: The significant impact of coronavirus disease 2019 (COVID-19) measures on elite and semi-elite South African athletes | J Sci Med Sport | 2020 |
| Ross R, et al. | Return-to-Play Considerations After COVID-19 Infection in Elite Athletes | J Athl Train | 2021 |
| Santos-Ferreira D, et al. | TEAM to Defeat COVID-19: A Management Strategy Plan to Address Return to Play in Sports Medicine | Orthop J Sports Med | 2020 |
| Savicevic AJ, et al. | Performance of Professional Soccer Players before and after COVID-19 Infection; Observational Study with an Emphasis on Graduated Return to Play | Int J Environ Res Public Health | 2021 |
| Schmidt T, et al. | Sports, Myocarditis and COVID-19: Diagnostics, Prevention and Return-to-play Strategies | Int J Sports Med | 2022 |
| Schwellnus M, et al. | Symptom cluster is associated with prolonged return-to-play in symptomatic athletes with acute respiratory illness (including COVID-19): a cross-sectional study-AWARE study I | Br J Sports Med | 2021 |
| Seligman E, et al. | Changes in training patterns and confidence to return to sport in United States collegiate athletes during the COVID-19 pandemic | Phys Sportsmed | 2022 |
| Snyders C, et al. | Symptom Number and Reduced Pre-infection Training Predict Prolonged Return to Training after SARS-CoV-2 in Athletes: AWARE IV | Med Sci Sports Exerc | 2022 |
| Stokes KA, et al. | Returning to Play after Prolonged Training Restrictions in Professional Collision Sports | Int J Sports Med | 2020 |
| Symanski JD, et al. | Myocarditis in the Athlete: A Focus on COVID-19 Sequelae | Clin Sports Med | 2022 |
| Tasca JS, et al. | Cardiac involvement in athletes infected by SARS COV-2 disease | Sci Sports | 2022 |
| van Hattum JC, et al. | Cardiac abnormalities in athletes after SARS-CoV-2 infection: a systematic review | BMJ Open Sport Exerc Med | 2021 |
| Vaudreuil NJ, et al. | Impact of COVID-19 on Recovered Athletes Returning to Competitive Play in the NBA "Bubble" | Orthop J Sports Med | 2021 |
| Wilson MG, et al. | Cardiorespiratory considerations for return-to-play in elite athletes after COVID-19 infection: a practical guide for sport and exercise medicine physicians | Br J Sports Med | 2020 |
| Woods S, et al. | Is a pandemic as good as a rest? Comparing athlete burnout and stress before and after the suspension of organised team sport due to Covid-19 restrictions, and investigating the impact of athletes' responses to this period | Psychol Sport Exerc | 2022 |
| Zhan H, et al. | COVID-19 countermeasures of Chinese national athletes: Prevention, treatment, and return to play | Sports Med Health Sci | 2022 |

Table S3. Risk of bias of missing items in data structure

| 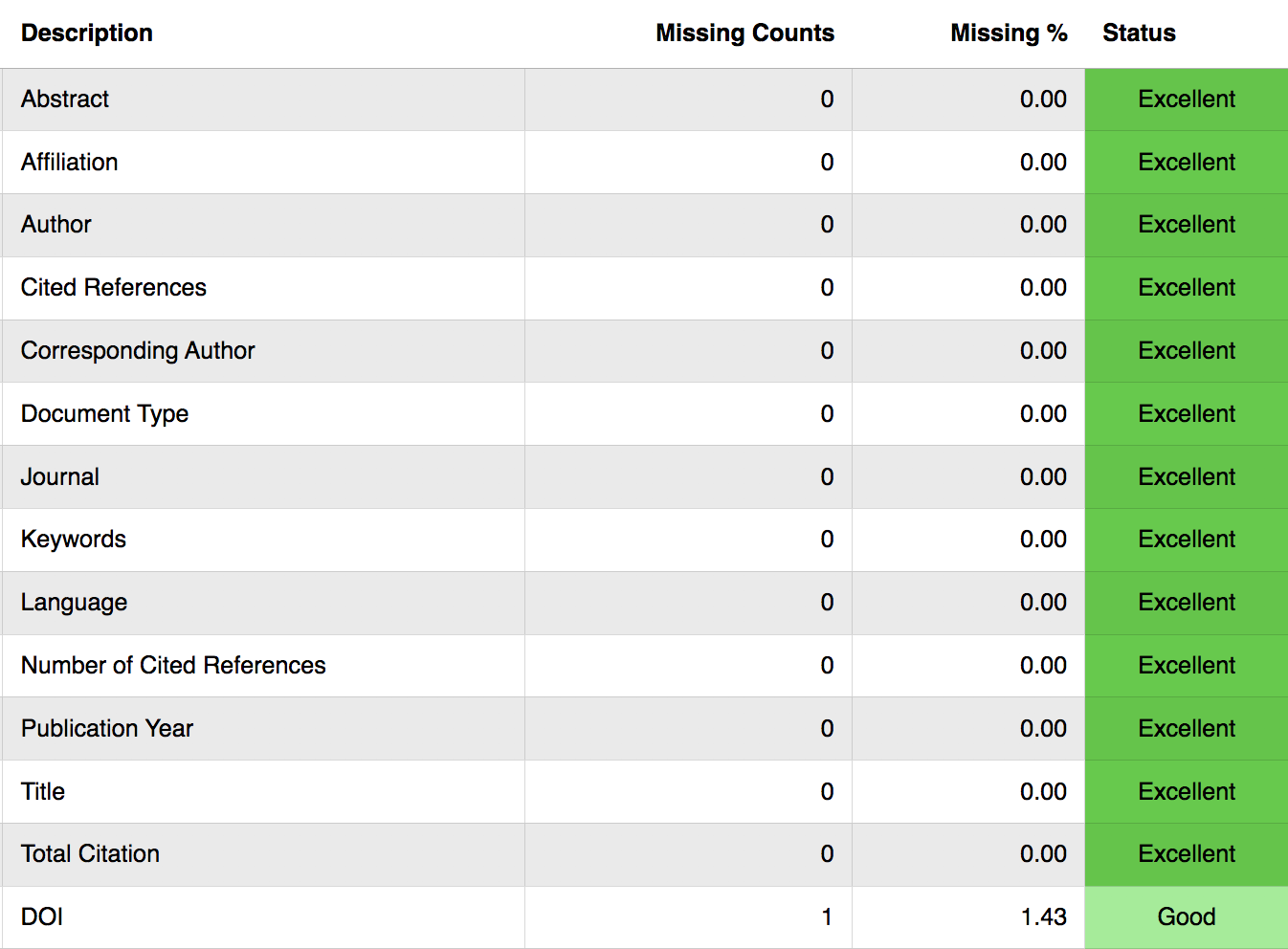 |
| --- |
